# *De novo* transcriptome of *Gymnema sylvestre* identified putative lncRNA and genes regulating terpenoid biosynthesis pathway

**DOI:** 10.1038/s41598-019-51355-x

**Published:** 2019-10-16

**Authors:** Garima Ayachit, Inayatullah Shaikh, Preeti Sharma, Bhavika Jani, Labdhi Shukla, Priyanka Sharma, Shivarudrappa B. Bhairappanavar, Chaitanya Joshi, Jayashankar Das

**Affiliations:** Gujarat Biotechnology Research Centre, Department of Science & Technology, Gandhinagar, 382011 India

**Keywords:** Sequence annotation, Plant molecular biology

## Abstract

*Gymnema sylvestre* is a highly valuable medicinal plant in traditional Indian system of medicine and used in many polyherbal formulations especially in treating diabetes. However, the lack of genomic resources has impeded its research at molecular level. The present study investigated functional gene profile of *G. sylvestre* via RNA sequencing technology. The *de novo* assembly of 88.9 million high quality reads yielded 23,126 unigenes, of which 18116 were annotated against databases such as NCBI nr database, gene ontology (GO), KEGG, Pfam, CDD, PlantTFcat, UniProt & GreeNC. Total 808 unigenes mapped to 78 different Transcription Factor families, whereas 39 unigenes assigned to CYP450 and 111 unigenes coding for enzymes involved in the biosynthesis of terpenoids including transcripts for synthesis of important compounds like Vitamin E, beta-amyrin and squalene. Among them, presence of six important enzyme coding transcripts were validated using qRT-PCR, which showed high expression of enzymes involved in methyl-erythritol phosphate (MEP) pathway. This study also revealed 1428 simple sequence repeats (SSRs), which may aid in molecular breeding studies. Besides this, 8 putative long non-coding RNAs (lncRNAs) were predicted from un-annotated sequences, which may hold key role in regulation of essential biological processes in *G. sylvestre*. The study provides an opportunity for future functional genomic studies and to uncover functions of the lncRNAs in *G. sylvestre*.

## Introduction

*Gymnema sylvestre* R.Br (Family, Asclepidaceae), also known as gurmar or Madhunashini, is a woody climber and a well-known highly valued medicinal plant used to treat diabetes in India since ages^1^. The leaves of *G. sylvestre* contain triterpene saponins belonging to oleanane and dammarane classes. The major constituents like gymnemic acids and gymnemasaponins are members of oleanane type of saponins while gymnemasides are dammarane saponins^2^. They are known for their antidiabetic, hypolipidemic^3^, stomachic, diuretic, refrigerant and astringent properties^1^. In addition, it is also known to exhibit anticancer activity^4^. Most importantly, gymnemic acids stimulate an antihyperglycemic response by regeneration of pancreatic cells, causing insulin release and inhibition of glucose absorption^5^. It is also known that *G. sylvestre* leaves not only produce blood glucose homeostasis but also increase uptake and activities of enzymes like phosphorylase, gluconeogenic enzymes and sorbitol dehydrogenase that are involved in glucose utilization via insulin dependent pathways^6^. In recent year’s, genomic profiling technologies such as RNA sequencing have emerged as effective tool in understanding functional genomics profile of non-model plants. RNA-seq has been used by scientific community in the identification of functional genes involved in the biosynthesis of active compounds and metabolic engineering of important pathways in plants^7–9^. Recent studies with *Curcuma longa*^10^, *Withania somnifera*^11^, *Camelina sativa*^12^, *Andrographis paniculata*^13^, *Solanum trilobatum*^14^, *Foeniculum vulgare*^15^ and *Arisaema heterophyllum*^16^ have demonstrated the effectiveness of *de novo* assembly of transcriptomes. Despite the importance of this plant, there is a dearth of data relating to its functional genomics profile except for a recent report describing polyoxypregnane glycoside biosynthesis pathway^17^. However, a detailed description of the expressed transcripts of *G. sylvestre* and putative genes involved in terpenoid biosynthesis pathways is still not known. In the present study, *de novo* transcriptome sequencing of *G. sylvestre* leaf was performed using Ion-Proton platform and analysis using various bioinformatics tools. The study will serve as a road to discover and decipher expression information like biosynthetic pathways and putative candidates of important secondary metabolites, which may be further used for the scale up production of bioactive compound.

## Materials and Methods

### Plant material and RNA isolation

Young and fully expanded leaves were collected in biological triplicates from disease-free one year old plants *Gymnema sylvestre*, grown at State Medicinal and Aromatic Plants garden, Gandhinagar, Gujarat, India and identified by State Medicinal Plant Board, Gujarat. The leaves were snap chilled immediately by dipping in liquid nitrogen and ground into fine powder using mortar and pestle. Further, total RNA was isolated according to manufacturer’s protocol using Qiagen Plant RNeasy isolation kit and RNase-free DNase I treatment was given in order to remove any traces of genomic DNA. For quality check, QIAxpert and QIAxcel were used for determining RNA integrity, while quantification was done using Qubit4.

### Generation of cDNA library and sequencing of transcriptome

Samples with RNA Integrity Score (RIS) values greater than 8.0 were further processed for preparing cDNA library. Ribosomal RNA depletion was carried out using RiboMinus RNA plant kit for RNA-Seq (Life Technologies, CA). The whole transcriptome cDNA library was prepared using Ion Total RNA-Seq kit V2 (Life Technologies Corporation, CA). Double stranded cDNA was ligated to barcoded adapters, loaded onto the Ion PI™ Chip (Ion torrent, Life technologies, CA) and sequenced in triplicate according to the standard protocol using Ion Proton System (Ion torrent, Life technologies, CA).

### *De novo* assembly of transcriptome

Sequencing data was collected and further, the raw reads were subjected to stringent filtering conditions for the removal of reads with adaptors, reads with ambiguous bases and reads with low quality using FASTX toolkit (http://hannonlab.cshl.edu/fastx_toolkit/). High quality (HQ) reads (i.e., each base having ≥20 phred score) were considered for assembling transcriptome. Primary assembly was carried out by merging the HQ reads using “Trinity” assembler^18^ with a minimum contig length of 200 bases and k-mer size of 25 bp. A minimum count of 2 k-mers were assembled by Inchworm algorithm and a minimum number of 5 reads were used to glue two Inchworm contigs together. In order to cluster contigs originating from the same gene or protein, a secondary assembly was carried out using CD-HIT EST (v4.6.1) tool^19^. Homologous contigs with 80% identity were clustered to generate full length transcripts. In order to determine the percentage of reads mapped to assembled transcriptome, we mapped the assembled transcriptome onto the processed reads using Bowtie2^20^. The resulting file was further used as input for eXpress tool to determine the FPKM and TPM values for the reads (https://pachterlab.github.io/eXpress/index.html).

The sequence data generated in this study have been deposited at NCBI in the Short Read Archive database under the accession number SRR7876667, SRR9644907, SRR9644908.

### Functional annotation and classification of transcripts

Assembled transcript sequences were functionally annotated using public databases. Sequence similarity search was performed using a BLASTX against the Uniprot and Swissprot databases and Pfam database using the Trinotate pipeline^18^. The Trinotate annotation pipeline includes several software packages such as BLASTX, BLASTP and PFAM search that are essential in transcriptome functional annotation. All analyses were performed in parallel using assembled FASTA sequences. Gene Ontology (GO) and Conserved Domain Database (CDD) were used to annotate the transcripts based on similarity. The GO analysis helps us in specifying all the annotated sequences comprising of GO functional group such as Biological Process, Molecular Function and Cellular Component^21^. Translated peptides were generated using the Transdecoder program embedded in the Trinity assembly pipeline for protein-based analysis using Eukaryotic Orthologous Group (KOG) classification. All results were deposited into Trinotate-provided SQLite database template and a spreadsheet summary report was generated from Trinotate using BLASTX E-value cutoff of 1e-5. Pathway assignment for the annotated transcripts was carried out using KEGG mapping (http://www.genome.ad.jp/kegg/). KEGG orthologs were identified using the KEGG Automated Annotation Server (KAAS) with default parameters. Transcripts were also annotated simultaneously using Function Annotator for transcriptome data^22^. FunctionAnnotator includes scripts and annotation tools, including LAST, BLAST2GO, PSORT, TMHMM, etc. for annotating GO terms, enzyme and domain identification, predictions for subcellular localization, lipoproteins, secretory proteins and transmembrane proteins, etc. FDR corrected GO terms were filtered and comparison with the closely related species were performed with similarity search E-value 10e-5.

### Identification of transcription factor families

Transcription factors (TFs) were identified using genome-scale protein and nucleic acid sequences by analyzing InterProScan domain patterns in protein sequences with high coverage and sensitivity using PlantTFcat analysis tool (http://plantgrn.noble.org/PlantTFcat/)^23^.

### Identification of simple sequence repeats (SSRs)

Simple sequence repeats were identified using MIcroSAtellite identification tool v1.0 (MISA) (http://pgrc.ipk-gatersleben.de/misa/). Unit size cut-off of six was used to consider a di-nucleotide repeat and 5 for SSRs of 3, 4, 5, and 6-nucleotide repeats. Maximum of 100 interrupting bases were allowed between two SSRS in a compound microsatellite.

### Prediction of long non-coding RNA (lncRNAs)

The non-coding DNA sequences (CDS) of *G. sylvestre* were used as the starting point for the prediction of lncRNAs. The CDS with length greater than 200 nucleotides^24^ were retained. The coding potential for the sequences were checked by Coding Potential Calculator (CPC), developed on support vector machine^25^. Based on CPC score (S), sequences were classified into non-coding (S ≤ −0.5), neutral (−0.5 < S < 1.0) and coding (S ≥ 1.0). The sequences were further searched using BLASTX against the SWISS-PROT database with an e-value cut-off of 0.001 in order to be sure that the sequences were non-protein coding. A database of lncRNAs was created using 45 plant species from the GreeNC^26^ and Blastn was performed. The sequences with more than 90% identity were predicted to be lncRNAs.

### Quantitative reverse transcription PCR (qRT-PCR) of selected secondary metabolite biosynthetic pathway genes in *G. sylvestre* leaf sample

qRT-PCR enables the detection and identification of target mRNA transcripts. Hence, to validate our dataset, some of the assembled *G. sylvestre* unitranscripts involved in Terpenoid biosynthetic pathway were selected for performing qRT-PCR. Total RNA from the leaves of *G. sylvestre* in biological triplicates was isolated from using Plant RNeasy isolation kit according to manufacturer’s protocol. cDNA was synthesized using Oligo(dT) and SuperScript III Reverse Transcriptase. Transcripts encoding squalene monooxygenase (SQLE), farnesyl-diphosphate farnesyltransferase (FDFT1) involved in Sesquiterpenoid and triterpenoid biosynthesis and 2-C-methyl-D-erythritol 2,4-cyclodiphosphate synthase (ispF), (E)-4-hydroxy-3-methylbut-2-enyl-diphosphate synthase (ispG), farnesyl diphosphate synthase (FDPS2) & diphosphomevalonate decarboxylase (MVD) involved in Terpenoid backbone biosynthesis were validated against house-keeping transcript Actin B, GAPDH, Beta-tubulin and Ubiquitin C as reference. Transcript specific primers were designed and PCR based expression profiling was carried out for each transcript in triplicates.

## Results

### Sequencing of cDNA library and *de novo* assembly of transcriptome

Sequencing of cDNA library using the Ion Proton generated millions of reads with an average length of 200 bp after the removal of adapter sequences and low quality reads with Phred score <20. 88.9 million of high quality data reads were obtained representing 85% of the transcriptome. Currently no reference genome is available for *G. sylvestre* therefore the Trinity assembler^20^ was used for *de novo* assembly of the high quality reads. Assembly of high quality reads using Trinity assembler produced a total of 23126 unigenes post removal of redundant transcripts using CD-HIT. Transcript length ranged from 200–7200 bp with an average of 369 bp and N50 of 372 bp was obtained. *De novo* assembly of transcriptome revealed 42.69% of GC content. The raw reads were mapped on to the assembly using Bowtie2 and 85% alignment was observed indicating good quality assembly.

### Functional annotation and classification of the clustered transcripts

**Figure 1:**
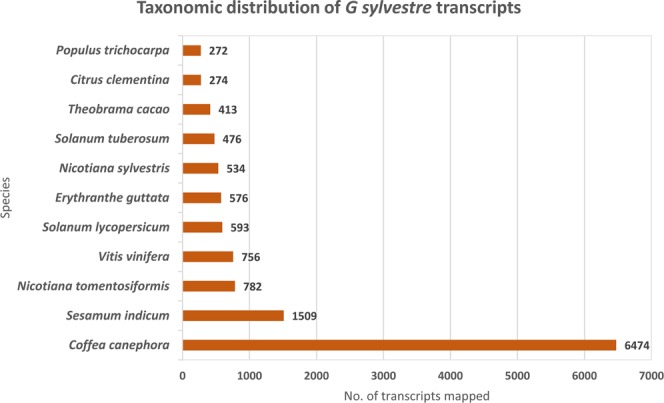
Taxonomic distribution of *Gymnema sylvestre* transcripts across plant nr database.

Extensive functional annotation was performed in order to decipher the profile and information regarding molecular functions, SSRs, transcription factors as well as signal peptides. Additionally, lncRNA were also predicted via *in silico* approach. Total 18116 unigenes were functionally annotated, whereas 5010 did not show similarity to any proteins or domains. Corresponding GO IDs were classified into biological functions, cellular components and molecular functions. Functional annotations of the assembled transcripts revealed that almost 52% of them showed homology to 11 other species. While the majority of them (35.73%) were homologous to the species *Coffea canephora* lowest homology was found with *Populus trichocarpa* (1.5%) (Fig. 1). The un-annotated unigenes show that there may be genus specific or species-specific functions.

### Annotation with gene ontology (GO)

**Figure 2:**
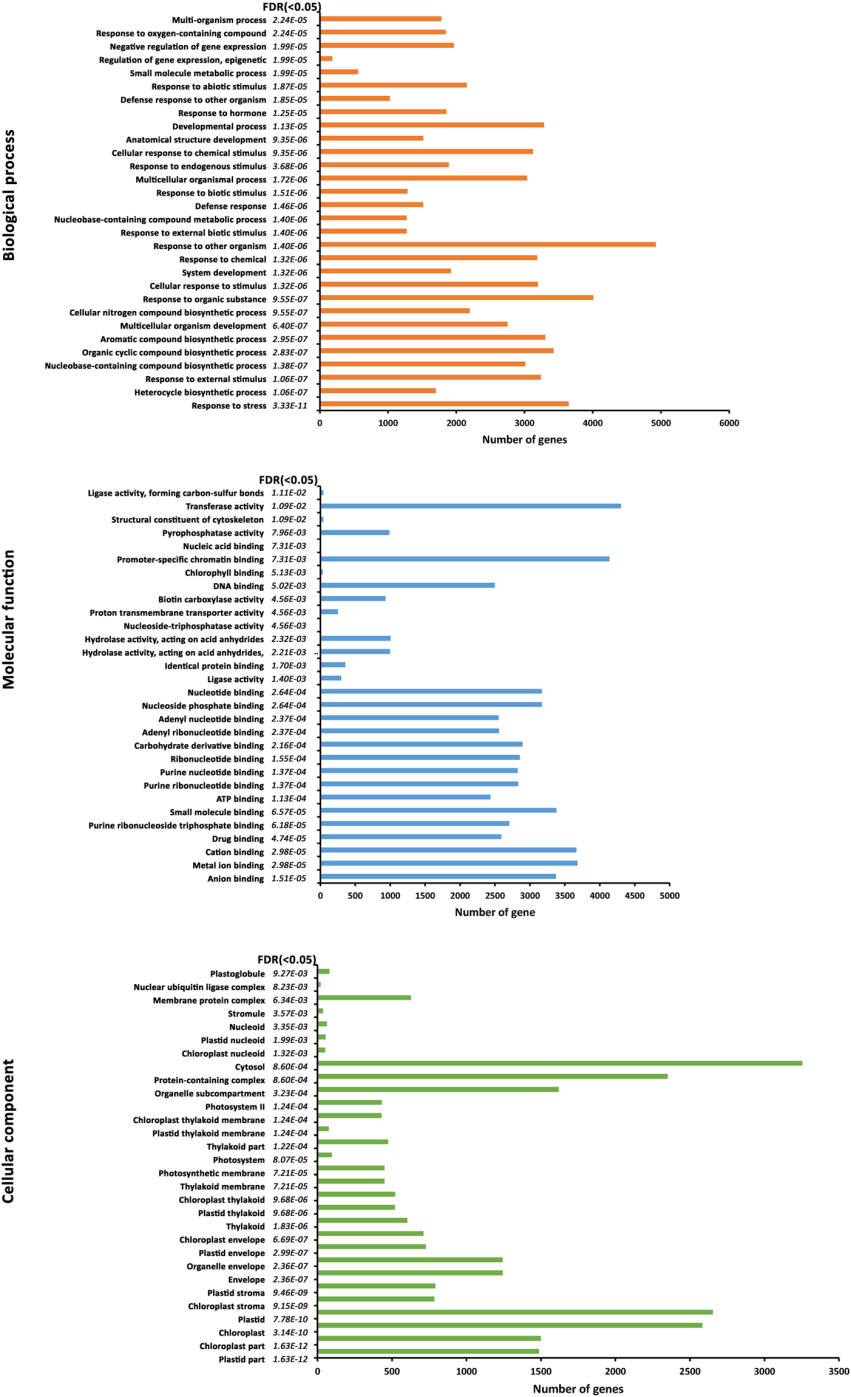
Top 20 GO enriched terms of transcripts in biological processes, cellular components and molecular function.

Out of 18116 annotated unigenes, 14987 were found to be associated with gene ontology terms. A total of 13085 unigenes were found in the category of biological processes with majority belonging to oxidation-reduction processes, metabolic processes, protein phosphorylation, response to cadmium ion, and regulation of transcription etc. (Fig. 2). About 12349 transcripts mapped to different molecular functions with majority of them belonging to ATP binding followed by zinc ion binding, DNA binding, protein serine/threonine kinase activity etc and 12690 transcripts mapped to cellular components belonging to membranes of nucleus, plasma membrane, cytosol, plasmodesma etc.

### Metabolic pathway analysis by KEGG

**Figure 3:**
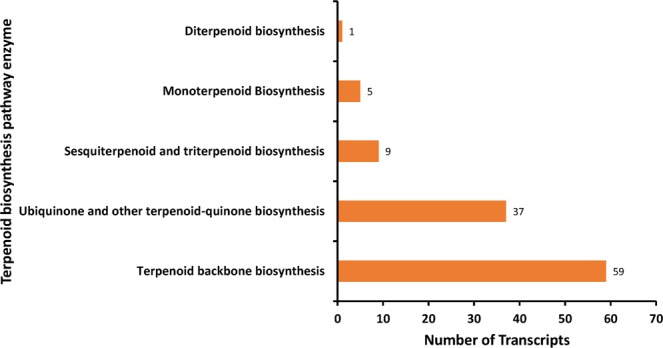
KEGG analysis showing number of transcripts mapped to enzymes involved in terpenoid pathways.

**Figure 4:**
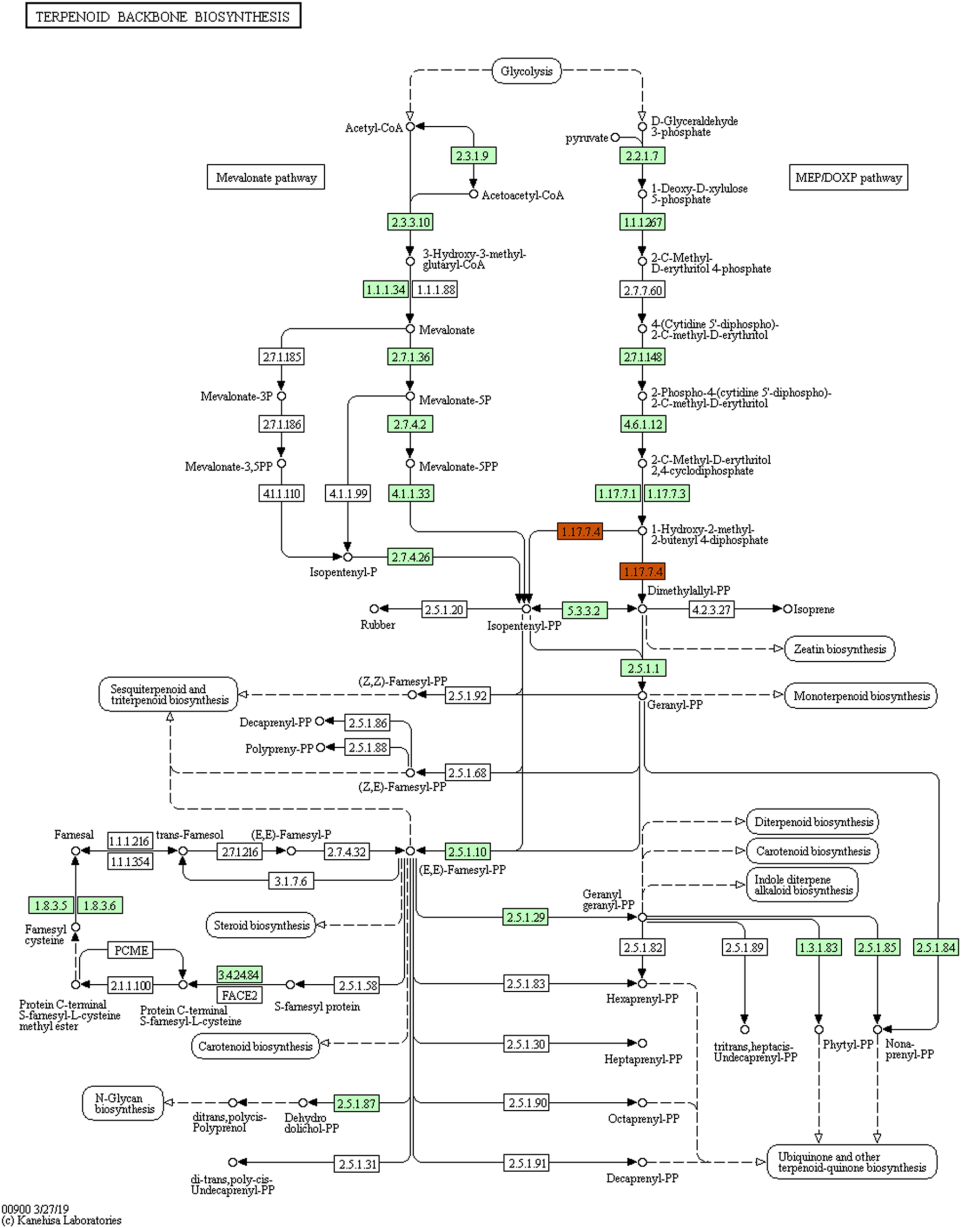
Transcripts mapped on the Terpenoid Biosynthetic pathway (Enzymes highlighted in one colour code for one enzyme. Green colour depicts different enzyme code). KEGG pathway map 00900 is mined here from http://www.kegg.jp/kegg/kegg1.html. The KEGG database has been reported previously^55–57^.

**Figure 5:**
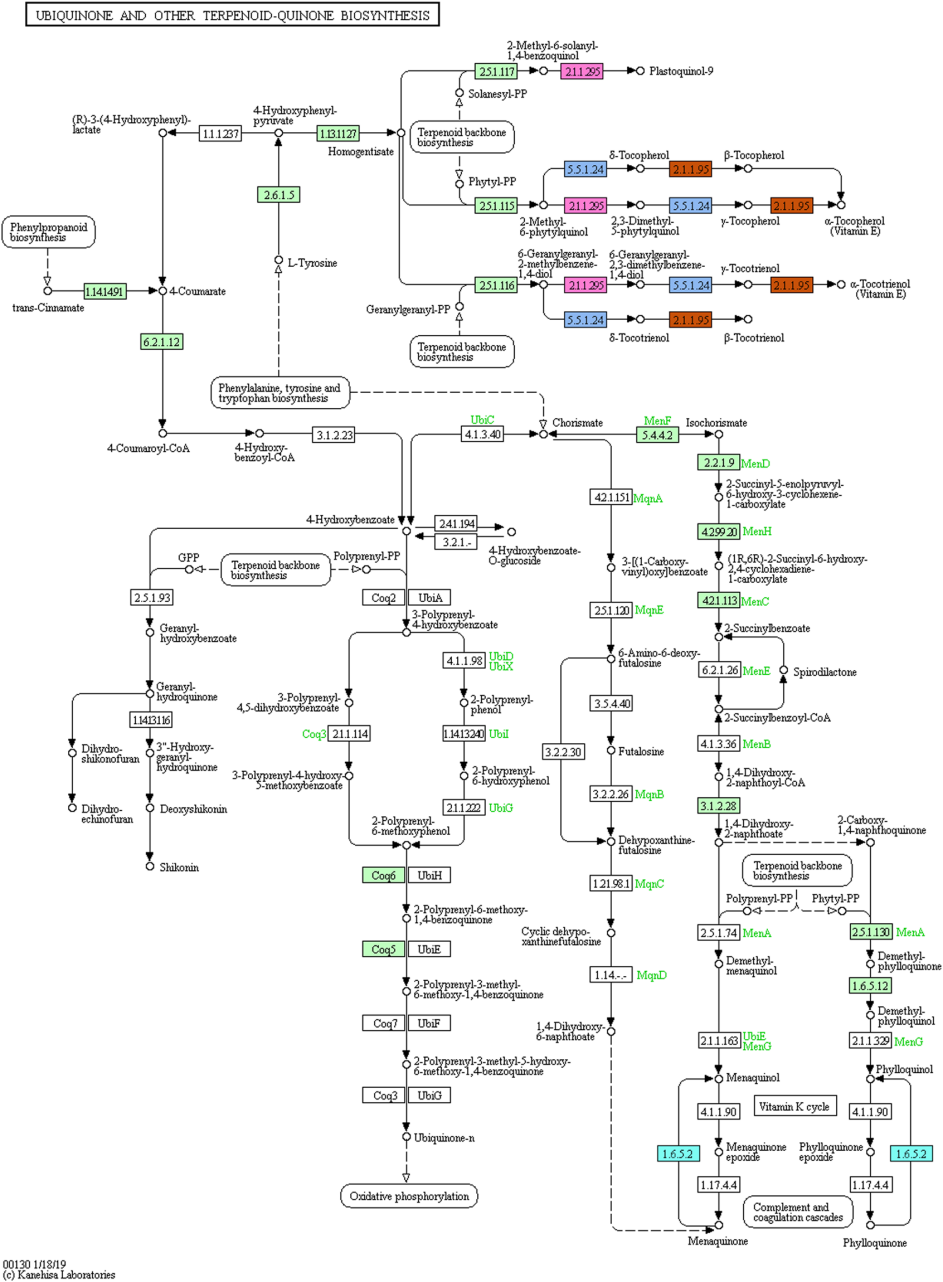
Transcripts mapped on ubiquinone and other terpenoid-quinone biosynthesis (Enzymes highlighted in one colour code for one enzyme. Green colour depicts different enzyme code). KEGG pathway map 00130 is mined here from http://www.kegg.jp/kegg/kegg1.html. The KEGG database has been reported previously^55–57^.

**Figure 6:**
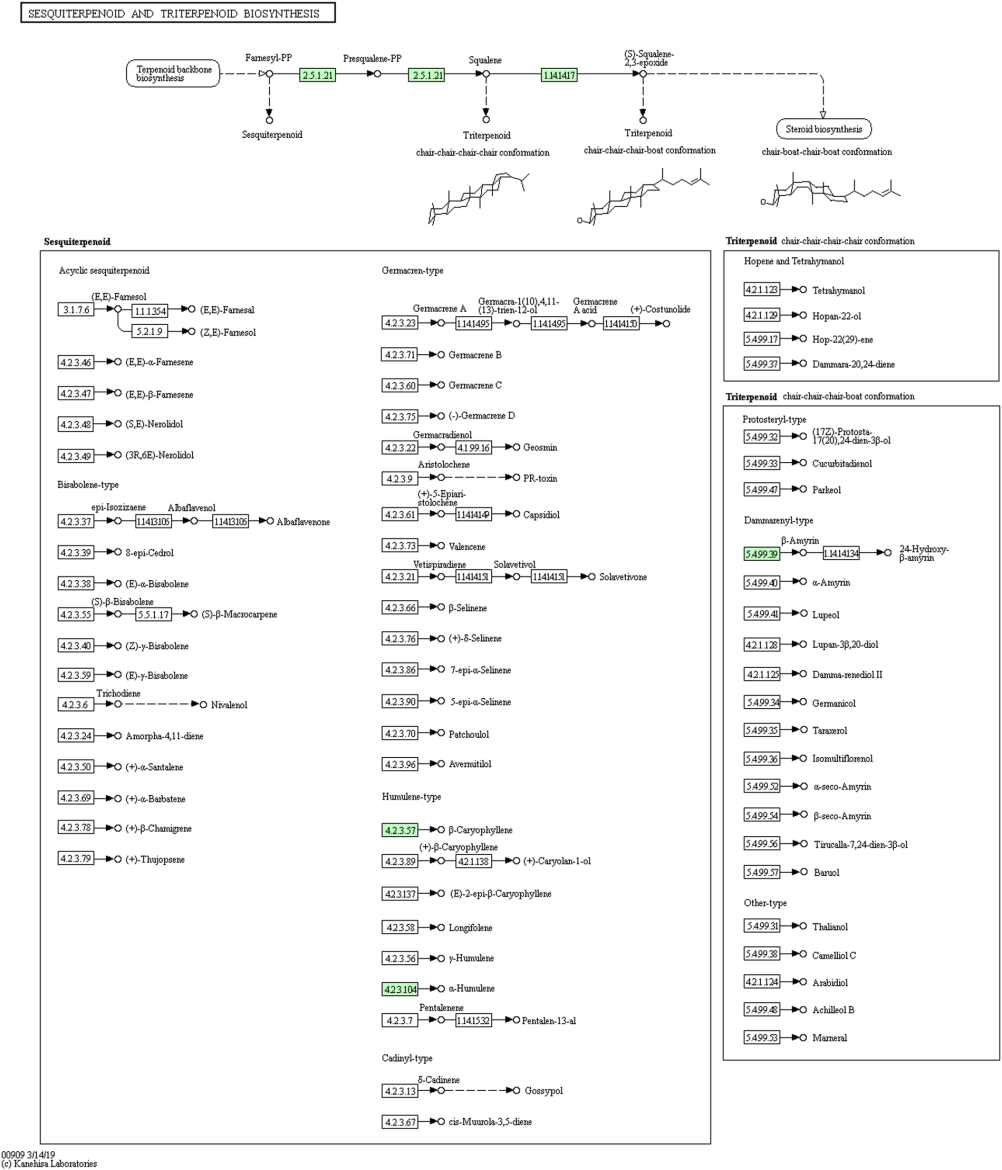
Transcripts mapped on biosynthesis of sesquiterpenoid and triterpenoid biosynthesis pathway (Enzymes highlighted in one colour code for one enzyme. Green colour depicts different enzyme code). KEGG pathway map 00909 is mined here from http://www.kegg.jp/kegg/kegg1.html. The KEGG database has been reported previously^55–57^.

**Figure 7:**
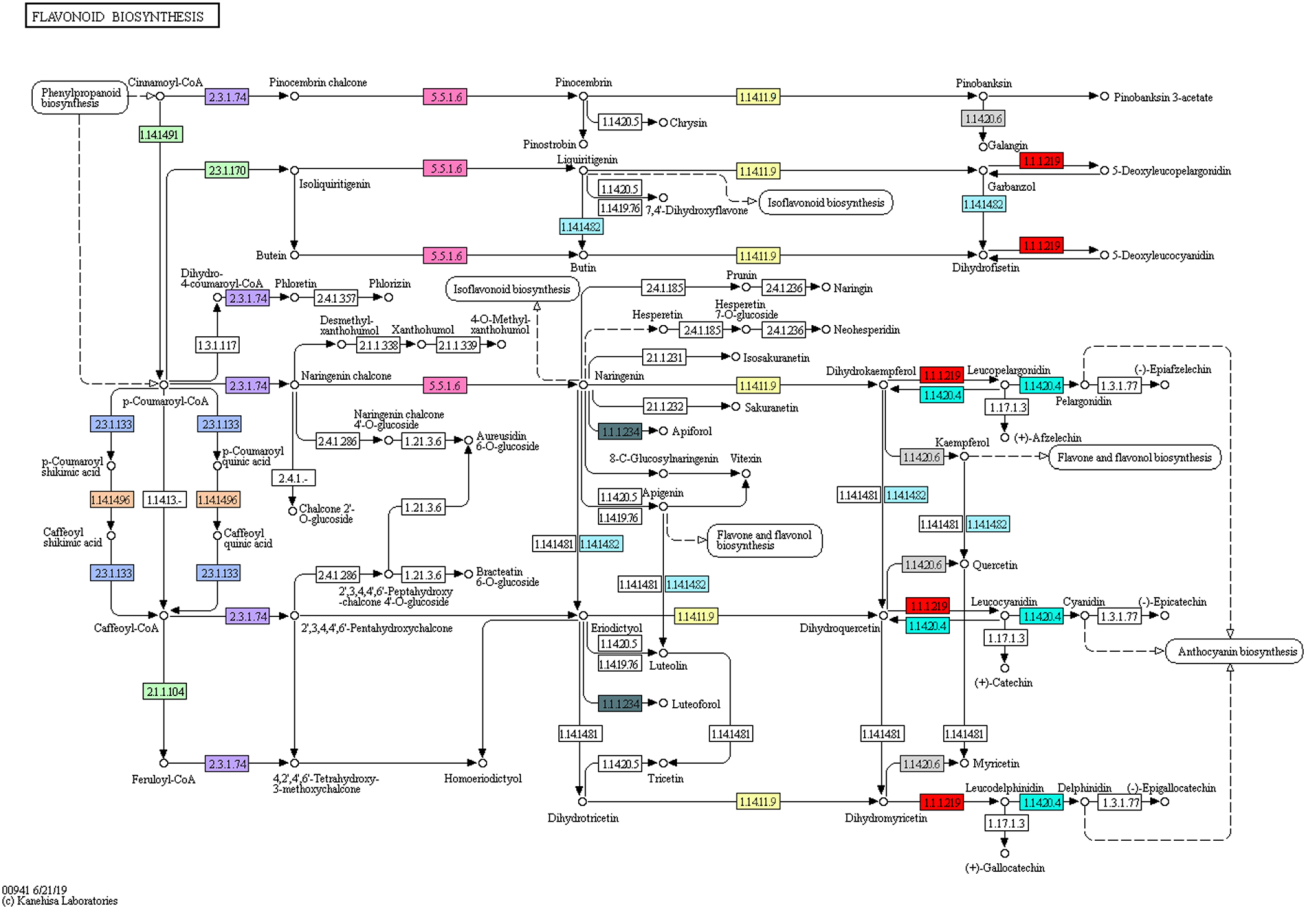
Transcripts mapped on the flavonoid biosynthetic pathway (Enzymes highlighted in one colour code for one enzyme. Green colour depicts different enzyme code). Green colour depicts different enzyme code). KEGG pathway map 00941 is mined here from http://www.kegg.jp/kegg/kegg1.html. The KEGG database has been reported previously^55–57^.

Present study annotated the leaf transcriptome of *G. sylvestre* and focused primarily on the terpenoid pathway unigenes. Identification of candidate genes and key enzymes are crucial in understanding the biosynthetic pathways of functional terpenoids in *G. sylvestre*. As pharmaceutical properties of *G. sylvestre* is largely dependent on its terpenoid profile, the present study was mainly focused on the identification of transcripts involved in terpenoid biosynthesis. The KEGG predictions of the present study mapped 111 transcripts encoding for various enzymes involved in the biosynthesis of different isoprenoids such as mono-terpenes, di-terpenes, tri-terpenes, and ubiquinones (Fig. 3). Analysis of transcripts involved in the terpenoid and diterpenoid biosynthetic pathways identified majority of them being involved in terpenoid biosynthesis followed by ubiquinone and other terpenoid-quinone biosynthesis (Figs 4–6). It was observed that the transcripts involved in Vitamin E synthesis, beta-amyrin synthesis and squalene synthesis were also mapped on the pathway as evident from Figs 5 and 6. Pathway analysis also showed 32 transcripts involved in the flavonoid biosynthesis pathway such as chalcone synthase, naringenin 3-dioxygenase, flavonoid 3′-monooxygenase, shikimate O-hydroxycinnamoyltransferase etc as depicted in Fig. 7.

### Identification of Transmembrane proteins, signal peptides, subcellular localization and CYP450

Analysis of the transcript sequences revealed 293 transcripts to have at least one enzyme hit. Total 4075 transcripts were identified to have at least one domain with >50% coverage (Supplementary Fig. S1). Total 2163 transcripts were predicted to have at least 1 transmembrane domain, whereas 541 transcripts were predicted to have signal peptides (Supplementary Table 1). In our present study total 39 transcripts which showed homology to CYP450 sequences.

### Transcription factor (TF) analysis and identification of SSRs

**Figure 8:**
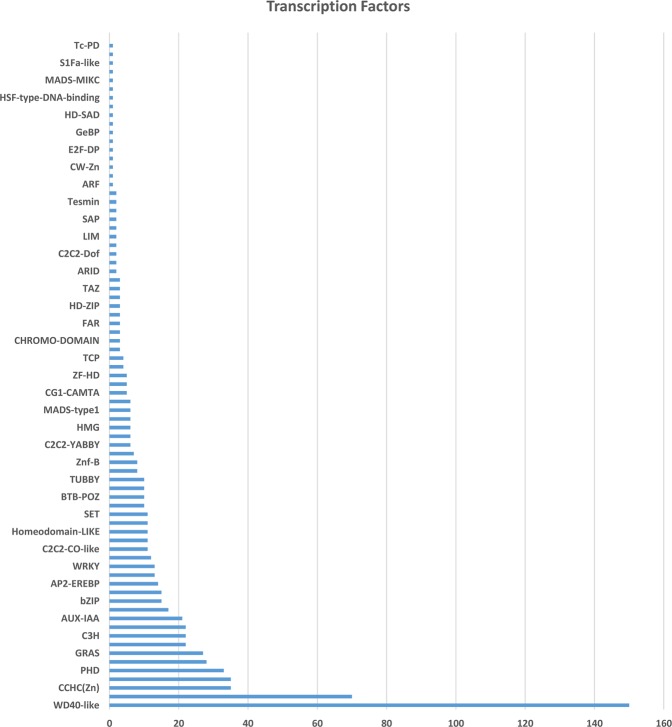
Transcription factor families detected from *Gymnema sylvestre* leaf transcriptome.

The analysis of transcripts revealed 809 unique transcripts belonging to 78 transcription factor families (Fig. 8). Among the identified unigenes, most of them represent WD40 family followed by C2H2, CCHC(Zn), Hap3/NF-YB, PHD etc. MISA analysis of 23126-clustered transcripts revealed a total number of 1428 SSRs in 1262 transcripts. More than 1 SSR was found in 135 transcripts including 96 transcripts with compound SSRs. A maximum number of SSRs were identified as di-nucleotide repeats followed by tri-nucleotide, mono-nucleotide, tetra-nucleotide, and penta-nucleotide repeats.

### Identification of long non-coding RNA (lncRNAs)

The 5010 un-annotated sequences were considered for predicting lncRNA. The coding potential of non-coding transcripts was determined using Coding Potential Calculator (Supplementary Table 2). Coding potential calculator provides coding probability, isoelectric points and fickett scores for the transcripts and gives a probability whether or not the transcript may be coding or non-coding. Transcripts having CPC score <0.2 were considered as non-coding. A database of lncRNAs was created using 45 plant species from the GREENC and Blastn was performed. Total 8 putative lncRNA were predicted from 2 plant species, in 10 *Gymnema sylvestre* transcripts (Table 1). Majority of predicted lncRNA were from *Arabidopsis lyrata* (932008, 932001, 931993, 484743, 932003, 930998) followed by from *Ananas comosus* species. These candidate sequences were searched in the Phytozome database^27^ and PANTHER database^28^ to determine whether any functional role was reported for the homologous sequences.

**Table 1:** LncRNA identified using GREENC database.

Transcript ID	Length (bp)	lncRNA ID	Homologous species
TRINITY_DN6221_c10_g1_i2	435	lcl|Alyrata_932008	*Arabidopsis lyrata*
TRINITY_DN6279_c0_g1_i1	213	lcl|Alyrata_932001	*Arabidopsis lyrata*,
TRINITY_DN6281_c64_g2_i1	533	lcl|Alyrata_931993	*Arabidopsis lyrata*
TRINITY_DN7807_c0_g1_i1	214	lcl|Acomosus_Aco027386.1	*Ananas Comosus*
TRINITY_DN8359_c0_g1_i1	261	lcl|Alyrata_484743	*Arabidopsis lyrata*
TRINITY_DN8360_c51_g1_i2	429	lcl|Alyrata_932003	*Arabidopsis lyrata*
TRINITY_DN8441_c0_g1_i2	381	lcl|Alyrata_930998	*Arabidopsis lyrata*
TRINITY_DN8483_c4_g1_i2	250	lcl|Acomosus_Aco028242.1	*Arabidopsis lyrata*

### Validation of transcripts using qRT-PCR

In order to validate the relative expression levels from the transcript abundance estimation, six important transcripts related to terpenoid, sesquiterpenoid biosynthetic pathways were chosen for RT-qPCR. The primer sequences for the transcripts designed as shown in Table 2. The nucleotide sequences for the same are provided in Supplementary Table S3. The relative expression of these transcripts was calculated using equation provided in Saussoy *et al*.^29^. Actin B, GAPDH, Ubiquitin C, beta-tubulin were chosen as reference for calculation of dCT. The expression of the transcripts have been provided in Supplementary Fig. S2. The expression of transcripts with individual housekeeping genes as reference are provided in Supplementary Fig. S3.

**Table 2:** List of Primers for qRT-PCR.

Gene	Name		Sequence
FDFT	farnesyl-diphosphate farnesyltransferase	Forward	AGAGGCGTGGTGAAATGAGA
		Reverse	TTGGCAGAGAGGTAGGCAAG
ispF	2-C-methyl-D-erythritol 2,4-cyclodiphosphate synthase	Forward	CAGCCAAAGAAGTCGCGATG
		Reverse	GGAAAGCCTCCGTTGAGACA
ispG	(E)-4-hydroxy-3-methylbut-2-enyl-diphosphate synthase	Forward	CCGGGTCCAGAACTGTTGAA
		Reverse	ATCCGAACGATGTCAGCTCC
FDPS	farnesyl diphosphate synthase	Forward	ATCTCCGATCTGCGAACCAC
		Reverse	TGTAGTCCAGCATCCGCTTG
MVD	diphosphomevalonate decarboxylase	Forward	GCTTTGGAACCACTTCCGCT
		Reverse	AGTGGAATGCGTGAGACAGT
SQLE	squalene monooxygenase	Forward	GGTTTGCTCACCTTGCGGAG
		Reverse	CAGGCCTTTACAAGATCTGCAC

## Discussion

In the present study, we performed leaf transcriptome sequencing and reported *de novo* assembly of *Gymnema sylvestre*. The *denovo* assembly of *G. sylvestre* resulted, 23,126 unigenes with an N50 of 372 bp and 42.69% of GC content. The quality of assembly based on N50 of unigenes, were near to the earlier transcriptome studies i.e. *Camellia sinensis*^30^ and *Rubber tree*^31^. These assembled unigenes and 85% alignment of reads onto assembled unigenes indicated good assembly quality. Functional analysis of the transcriptome annotated and classified 18116 unigenes into different biological processes, molecular functions and cellular components. The un-annotated unigenes show that there may be genus specific or species-specific functions.

Plant secondary metabolites have significant use in the food and pharmaceutical industries, which makes the study of biosynthesis, regulation and metabolic engineering of valuable secondary metabolites extremely useful^32,33^. Earlier report on *G. sylvestre* transcriptome indicated the synthesis of bioactive gymnemic acid takes place primarily in the leaf ^34^. Identification of candidate genes and key enzymes is crucial in understanding the biosynthetic pathways of functional terpenoids in *G. sylvestre*. As pharmaceutical properties of *G. sylvestre* largely depend on its terpenoid profile, the present study was mainly focused on the identification of transcripts involved in terpenoid biosynthesis. KEGG analysis mapped 111 transcripts encoding for various enzymes involved in the biosynthesis of different isoprenoids such as mono-terpenes, di-terpenes, tri-terpenes, and ubiquinones.

Precursor molecules for terpenoid biosynthesis are derived from the cytosolic mevalonate (MVA) and plastidial methyl-erythritol phosphate (MEP) pathways. Transcripts mapped on both MVA and MEP pathways which was evident from the data analysis. The results correlate with the hypothetical pathway provided by Tiwari *et al*.^1^ for gymnemic acid biosynthesis. We found many transcript genes related to isoprenoid biosynthesis from the MEP pathway including gene transcripts such as 1-deoxy-D-xylulose-5-phosphate synthase, 1-deoxy-D-xylulose-5-phosphate reductoisomerase, 4-(cytidine 5′-diphospho)-2-C-methyl-D-erythritol kinase, 2-C-methyl-D-erythritol 2,4-cyclodiphosphate synthase, (E)-4-hydroxy-3-methylbut-2-enyl-diphosphate synthase, isopentenyl-diphosphate Delta-isomerase and geranyl-diphosphate synthase. These transcripts were also validated via qRT-PCR and represented positive involvement in terpenoid biosynthesis via the MEP pathway. *G sylvestre* is known to produce atleast 34 different compounds including Vitamin E, squalene, beta-amyrin and related glycosides^35^. Pathway analysis also showed transcripts involved in synthesis of Vitamin E which is considered as an important free radical scavenger involved in the prevention of prostate cancer^35^. Apart from Vitamin E, transcripts were also found for (3S)-2,3-Epoxy-2,3-dihydrosqualene also known as Beta-amyrin synthase which is an enzyme that catalyzes the reaction to form beta-amyrin. Beta-amyrin is known to exhibit anti-inflammatory, anti-microbial activities^36^. Besides this, transcripts involved in flavonoid synthesis pathway were also found. *G. sylvestre* is also known to exhibit wound healing properties which may be attributed to the free radical scavenging action and presence of flavonoids^20,37^.

Transcription factors (TFs) play a major role in plant development and their response to the environment. The Transcription factors identified in the present study showed presence of WD40 like and CCHC as major transcription factor families in the transcriptome. Members of WD40 superfamily are increasingly being recognized as key regulators of plant-specific developmental events^38^ whereas CCHC (Zn) also known as transcription factor interactor and regulator specifically interact with single-stranded DNA or RNA oligonucleotides carrying recognition sequences^39^. Another transcription factor, which was abundant, was the plant homeodomain (PHD) which has been termed as an epigenome reader. PHD zinc fingers are known to be conserved and modify chromatin as well as mediate molecular interactions in gene transcription^40^.

Apart from gene discovery, transcriptome sequencing has also been proven to be an important tool for molecular marker development^41^. SSRs also known as microsatellites, are short repeating sequences with a unit size of mono-, di-, tri-, tetra-, or penta-nucleotides. In terms of the types of motifs found in SSR loci other than the mono- and large sized repeats, we found similar results as in previous report with plant microsatellites^42^. The most common tri-nucleotide repeats found were GAA/TTC, GAT/ATC, TCT/AGA and CAG/CTG. Interestingly, the proportions of di- and tri-nucleotide repeats were quite close (38.86% versus 34.87%) as reported earlier^43^.

In recent years, the functional characterization of one of the largest gene families, i.e. CYP450s, has created immense interest in the scientific community. They were known to catalyze the oxidative modification of various substrates using oxygen and NAD(P)H^44^. Many studies focusing on the transcriptome-wide identification of CYP450s for terpene biosynthesis have been reported^45,46^. An earlier research performed transcriptomic analyses based on 454 pyrosequencing data of *Panax ginseng* flowers, roots, stems, and leaves, which identified 326 potential CYP450s, including CYP716A47, which is related to the ginsenoside biosynthesis^47^. The current study identified 39 transcripts exhibiting homology to CYP450 sequences which may be of further interest to understand the involvement for the targeted biosynthetic pathway. Analysis of domains showed presence of a high number of transcripts for kinases like protein kinase-like domains, serine/threonine kinases, tyrosine-protein kinase etc. This suggests that most of the transcripts may be involved in signaling and regulatory processes, which correlates with our functional analysis.

With the advancements in sequencing technologies and high-throughput analysis tools the traditional view that protein-coding genes are the only effectors of gene function has been challenged. Micromolecules such as long noncoding RNAs (lncRNAs), miRNA etc. have been identified as key regulatory cascade of the eukaryotic transcriptomes, involved in the regulation of important biological processes in plants^48–50^ as well as in cross-kingdom gene regulation^51–53^. The study predicted 8 putative candidate lncRNA sequences using computational screening against database of 45 Plants species. Although some sequences showed annotation for the locus in PANTHER database^28^ no specific function was provided for these sequences, which may be due to the lag in lncRNA research in plants as compared to that in humans and animals^54^.

## Conclusion

In summary, our findings give a molecular insight of the transcriptome profile of an important antidiabetic medicinal plant. Due to its bioactive principle and potential use in Indian system of medicine through many polyherbal formulations, our study will enrich the understanding of the biosynthesis of its active principle. Our data provides us a glimpse of the transcripts, involved in secondary metabolic pathways. The transcriptome profile reveals the terpenoid, flavonoid and other secondary metabolic pathway genes, which adds information to *G. sylvestre* dataset and may help in accelerating the design-build-develop approach in metabolite engineering. Further, qRT-PCR results confirmed expression of a few selected transcripts proving the reliability of our *G. sylvestre* transcriptome study. Additionally, identified putative lncRNAs in the present study may further be explored in future experimental studies to uncover their role in regulation of various biological process in *G. sylvestre*. Such study on non-model plants will be of great potential in scaling up the targeted metabolite for therapeutic purposes.

## Supplementary information

Supplementary Information

Supplementary Table S1

Supplementary Table S2
