# Supplementary material for: De novo transcriptome of Gymnema sylvestre identified putative lncRNA and genes regulating terpenoid biosynthesis pathway
Source: Sci Rep. 2019 Oct 16;9:14876. doi: 10.1038/s41598-019-51355-x (PMC6795813; doi:10.1038/s41598-019-51355-x)
Supplement: Supplementary file 1 — Supplementary Information [file 41598_2019_51355_MOESM1_ESM.pdf]

Supplementary Information

De novo transcriptome of *Gymnema sylvestre* identified putative lncRNA and genes regulating terpenoid biosynthesis pathway

Garima Ayachit, Inayatullah Shaikh, Preeti Sharma, Bhavika Jani, Labdhi Shukla, Priyanka Sharma, Shivarudrappa B. Bhairappanavar, Chaitanya Joshi, Jayashankar Das*

Gujarat Biotechnology Research Centre, Department of Science & Technology, Gandhinagar - 382011, India.

***Corresponding Author's Email id: jayshankardas@gmail.com**

Figure S1: Pi chart represents distribution of transcripts across InterPro domains

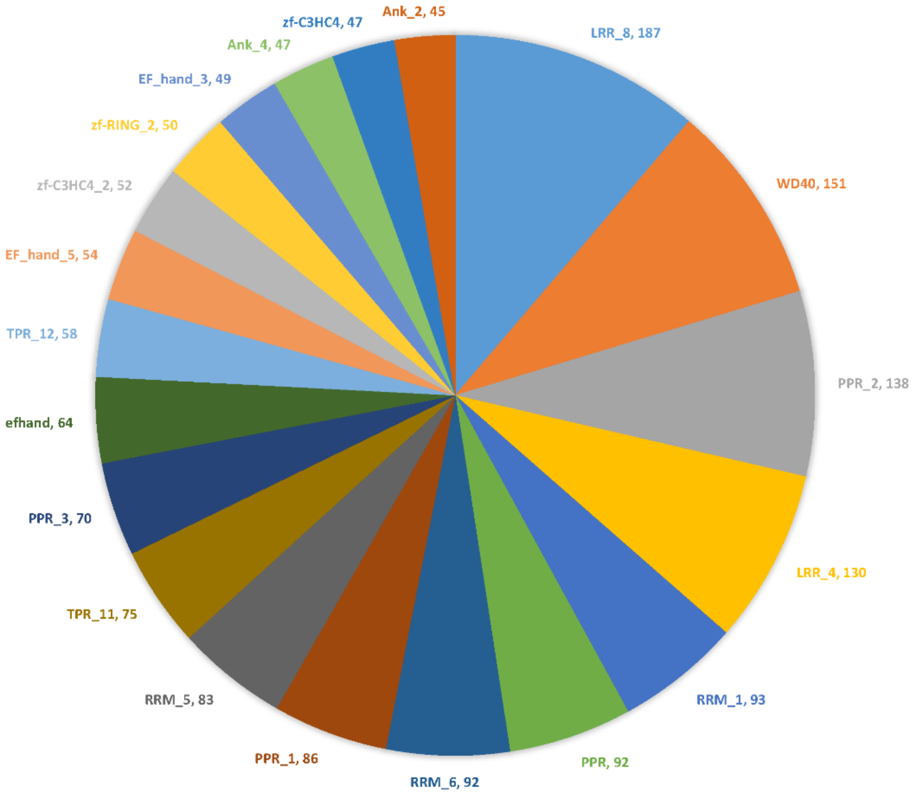

Figure S2: dCT values of the genes involved in Terpenoid biosynthetic pathway

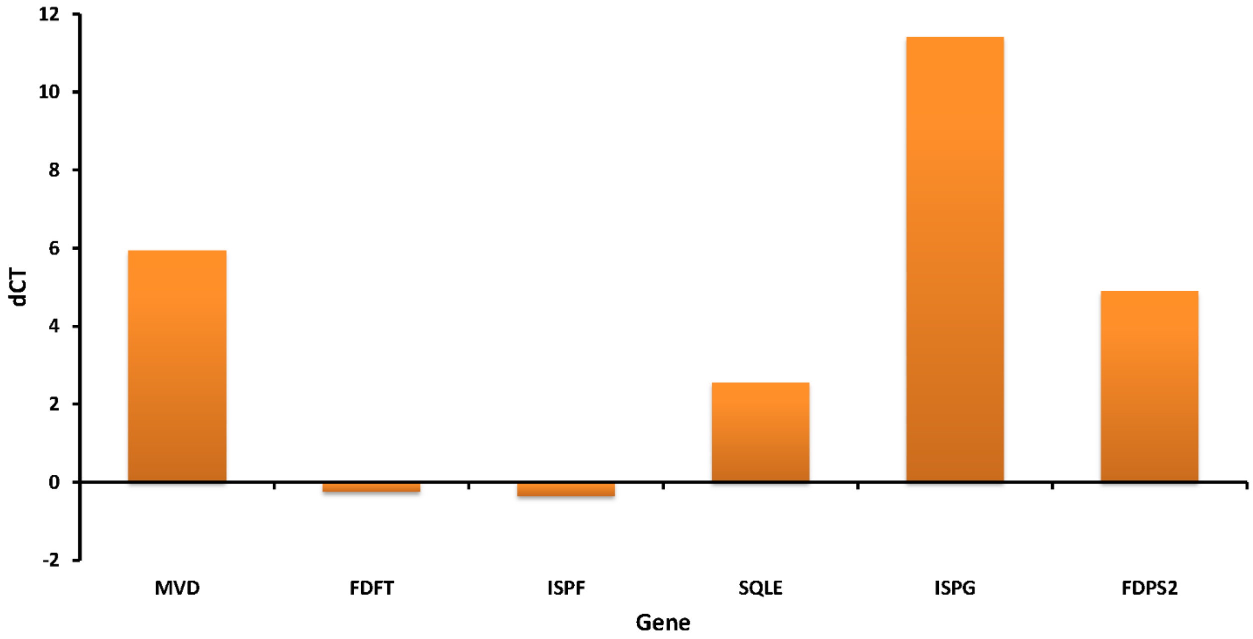

Figure S3: Expression data of genes against individual housekeeping genes

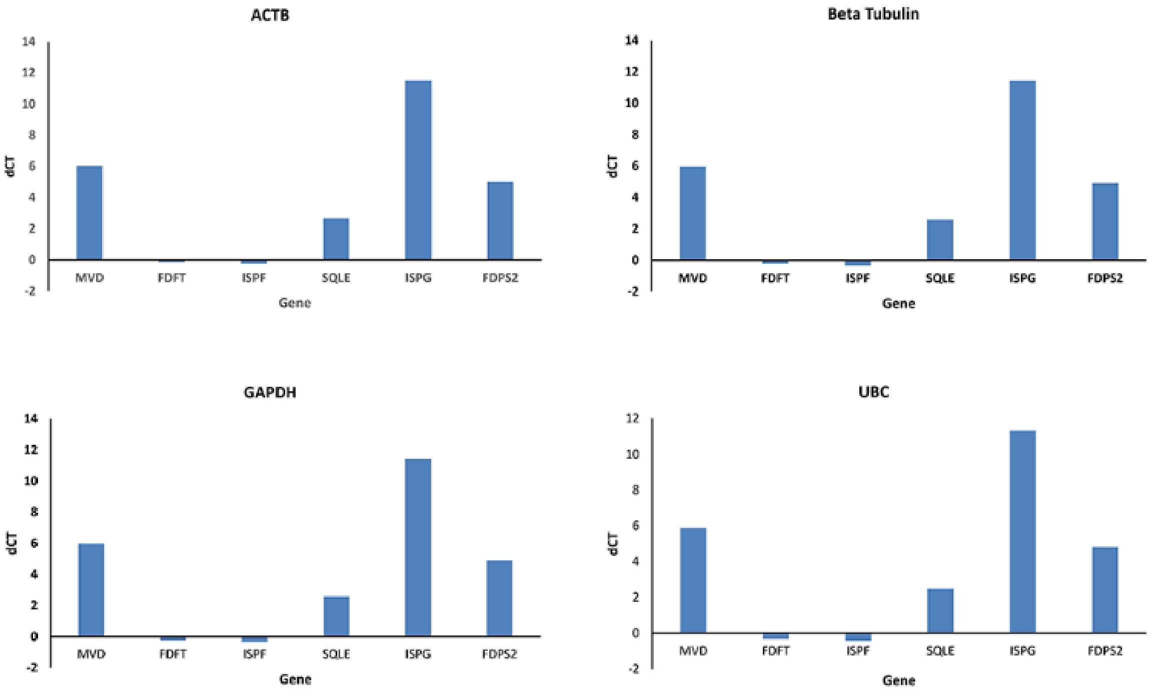

Supplementary Table S3: Sequences of Transcripts validated by qPCR

FDFT1<br>Farnesyl-diphosphate farnesyltransferase	AGAGGCGTGGTGAAATGAGACGTGGTCTTACTGCTAAAGTTATCGACAGAACCAA<br>AACCATGTCAGATGTCTATGGTGCATTTTTGATTTCTCATGTATGCTGATGTCTAAGGTT<br>GATGACAATGATCCTAATGCTAAGAAACTAAGAACAGGCTCGAAGCAATATTGAAATTT<br>G<br>CAGGGAATCTGGGACCCTACTAAAAGGAAATCCTACATAATGAGAACTGAGCCTGCATA<br>C<br>AACACTACAATGATTGTTTTCTTCCTTATTATTCTGGGCATTCTCCTTGCCTACCTCTCT<br>GCCAA
ispF<br>2-C-methyl-D-erythritol 2,4-cyclodiphosphate synthase	CAGCCAAAGAAGTCGCGATGGCCATGGCGGTTTCCCTCTCCTGCTCCACTTCATTTCTG<br>AGTAGAACGAAGCAAAATCTAGAGAATGTTCTTTGTTCTCGTACATGTCTCAACGGAGG<br>C<br>TTTCC
ispG<br>(E)-4-hydroxy-3-methylbut-2-enyl-diphosphate synthase	CCGGGTCCAGAACTGTTGAACTCCAGCCTGCATCAGAAGGAAGCCAGCTTTTAGTTCCTG<br>TTCAAAATACTGTGAATCCGTAC<br>ACAAAACTGTGAGGAGGAAAACCTGCACAGTTATGGTTGGGAATGTGGCCATTGGTAGT<br>G<br>ACCATCCCATTCGAATTCAAACAATGACAACGACGGACACTAAAGATGTTGCTGCAACA<br>G<br>TTGAACAGGTAATAAGGATAGCTGACAAGGGAGCTGACATCGTTCGG
FDPS<br>farnesyl diphosphate synthase	ATCTCCGATCTGCGAACCACATTCTTGAAGGTCTACGATG<br>TTCTCAAATCTGAGCTCTTAAACGATCCTGCTTTTGAATGGACAGATGATTCTTACCAGT<br>GGGTCAAGCGGATGCTGGACTACA
MVD<br>diphosphomevalonate decarboxylase	GCTTTGGAACCACTTCCGCTGCTCTATGTTGGATAAGAGGACTCGTTTCAACTGTCTCAC<br>GCATTCCACT
SQLE<br>squalene monooxygenase	GGTTTGCTCACCTTGCGGAGGGTGTAGAAGGATTCAAGATACTTGCACAAAGTAGATGC<br>ATCGTGCAGATCTTGTAAAGGCCTG
